# Capsule Endoscopy Transit Time to Duodenum: Relation to Patient Demographics

**DOI:** 10.7759/cureus.6894

**Published:** 2020-02-05

**Authors:** Alsadiq Al hillan, Diana Curras-Martin, Michael Carson, Shreya Gor, Adaeze Ezeume, Varsha Gupta, Albino Copcaalvarez, Gagan Beri, Mordechai Bermann, Arif Asif

**Affiliations:** 1 Internal Medicine, Jersey Shore University Medical Center, Neptune, USA; 2 Gastroenterology, Jersey Shore University Medical Center, Neptune, USA

**Keywords:** endoscopy, capsule endoscopy, gastric transient time by capsule endoscopy, esophagogastroduodenoscopy (egd) fasting time

## Abstract

Background

Anesthesia guidelines recommend fasting for at least two hours to minimize aspiration risk related to endoscopic procedures, and the American Society for Gastrointestinal Endoscopy (ASGE) states that the final oral preparation liquid can be administered three to eight hours before the procedure. We have observed the cancellation of endoscopy procedures if liquids were consumed within four, six, or eight hours of the start time. Objectively, documenting gastric transit time via a review of pill endoscopy data could address clinician concerns, prevent delays in patient care, and improve the rate at which our clinicians practice within national guidelines. The objective was to utilize capsule endoscopy data from our center to report the relationship between patient factors that could affect gastric transit time (GTT) and small bowel transit time (SBTT) such as chronic kidney disease (CKD), diabetes mellitus (DM), nutritional status, and obesity.

Methods

This retrospective review obtained data on adult pill endoscopy (PillCam™ SB 3) (Medtronic, Minneapolis MN) studies on in- and outpatients. Past medical history and laboratory data were abstracted from electronic medical records. Mean GTT and SBTT are reported in minutes + standard deviation (SD) and times were compared accounting for conditions that could prolong transit, such as diabetes mellitus or chronic kidney disease (CKD).

Results

One hundred and sixty-three records reviewed. Four patients were excluded as the pill did not pass out of the stomach. The mean age was 66 years, 57% were female, and 26% were evaluated for gastrointestinal (GI) bleeding. The mean GTT for all patients (n = 159) was 35 + 49 with a median of 19 minutes. There were no statistically significant differences in GTT between the following subgroups: CKD0 (n = 100) 40 + 58 versus CKD5 (n = 11) 35 + 39, albumin > 3.0 (n = 123) 37 + 53 versus albumin < 3.0 (n = 36) 27 + 30, diabetes mellitus (DM) (n = 40) 51 + 71 vs. non-DM (n = 119) 42 + 79, body mass index (BMI) > 30, or aspirin use. The SBTT results in all patients (n = 124) was 238 + 88 minutes. Similarly, there was no relation between SBTT and albumin, any CKD, CKD0 versus CKD5, DM status, or BMI. The patients with the capsule stuck in the stomach did not have any other clinical history to explain this occurrence.

Conclusions

This analysis of objective data regarding pill endoscopy found that the mean GTT was 44 minutes, and it was < 60 minutes for 85% of the cohort. Patient factors were not associated with longer transit times, and this is the first report to document PillCam times in relation to CKD. These data support recommendations that endoscopic procedures, in accordance with anesthesia and ASGE guidelines, can be safely conducted in the majority of patients within 60 minutes of ingesting liquids.

## Introduction

Gastrointestinal (GI) endoscopy is a widely used procedure requiring moderate sedation. In order to optimize visualization of gastric or intestinal mucosa and due to diminished airway-protective reflexes in response to anesthesia, the American Society of Anesthesiologists (ASA) guidelines recommend patients undergoing procedural conscious sedation should fast for six hours or more [1], but the American Society of Gastroenterology (ASGE) guidelines permit a clear liquid meal up to two hours before or a “light meal” up to six hours before anesthesia [2].

In adults, a fasting period of 1.5 to three hours compared to six hours did not increase the risk of aspiration [3] and was actually associated with decreased gastric volume [4-5]. In the pediatric population, the evidence does not support an increased risk of aspiration in patients who did not meet the ASA fasting recommendations versus those who did [6-14]. However, at our institution, we have observed multiple occasions where clinicians delay or cancel endoscopy cases if liquids were consumed within four and up to eight hours before the procedure, and some within shorter intervals if milk was consumed. These delays add to the inconvenience of being nothing per mouth (NPO) for many hours and may result in cancellation of the procedure, much to the frustration of the endoscopist and patient.

Objectively documenting gastric transit time (GTT) via a review of pill endoscopy data could address clinician concerns regarding actual transit time, prevent delays in patient care, and improve the rate at which our clinicians practice within national guidelines.

The objective was to utilize capsule endoscopy data from our center to report the relationship between patient factors that could affect GTT and small bowel transit time (SBTT), such as chronic kidney disease (CKD), diabetes mellitus (DM), nutritional status, and obesity.

## Materials and methods

This was a retrospective chart review of records from the capsule endoscopy center at the Hackensack Meridian Health - Jersey Shore University Medical Center, Neptune, NJ. Inclusion criteria were patients > 18 years of age who had a GTT study utilizing the PillcamSB3 video capsule endoscopic (VCE) device (Medtronic, Minneapolis, MN) recorded between 2012 and 2018 due to specific indications (Table 1). Studies ordered on outpatients included the clinical indication, age, and procedural vital signs. Studies obtained on inpatients allowed us to access to the hospital computer system and clinical notes for demographic data, including past medical history and laboratory values. The primary outcome was GTT obtained from the RAPID® software (Medtronic, Minneapolis, MN) stratified by patient conditions that could be associated with slower transit times and/or gastric/intestinal neuropathy: diabetes status (yes vs no), HbA1c > 6.5% vs. < 6.5%, CKD Stage 0 vs. Stage 5, CKD Stage 0 vs. Stages 2-5, albumin as a continuous variable, albumin as a dichotomous variable (< 3.0 mg/dL vs. > 3.0 mg/dL), body mass index (BMI) as a continuous variable, and obesity (BMI > 30 vs. < 30 kg/m^2^). Diabetes and chronic kidney disease may cause gastric neuropathy which slows transit time. Albumin levels may be low in patients who are malnourished, have a chronic medical disease, or are acutely ill, so we explored the theory that bowel wall edema due to low oncotic pressure could be associated with longer transit times. Medical conditions were classified based on documentation made by the providers within the clinical notes. GTT and SBTT were reported as mean (minutes) + standard deviation (SD) statistics: descriptive, Student’s t-test to compare means for parametric data, and Wilcoxon rank-sum test to compare means for non-parametric data, and Kruskal-Wallis test to analyze means of ordinal variables. Stata 15 (StataCorp LLC, College Station, TX) was used for analysis.

**Table 1:** Indication for Video Capsule Endoscopy (n)

Indication	Number
Anemia	132
Abdominal pain	17
Diarrhea	7
Inflammatory Bowel Disease	2
Weight Loss	2
Abnormal Imaging	2
Other	1
Total	163

The study was approved by the Hackensack Meridian Health Institutional Review Board (#201805302J).

## Results

One hundred and sixty-three records were reviewed. Table 2 lists the patient and subgroup demographics. The mean age was 66 years, 57% female patients, 26% evaluated for GI bleeding, the mean hemoglobin was 9.1 mg/dL + 3, and mean ferritin 195 + 395 mg/ml. The ages of the four patients with the capsule stuck in the stomach were 61, 66, 72, and 82, and none of these patients had diabetes, CKD, low albumin, or information in the chart suggesting the presence for risk factors for gastric outlet obstruction.

**Table 2:** Demographics BMI: body mass index; GI: gastrointestinal

	Mean ± Standard Deviation or %	# of Subjects
Mean Age (years)	66 ± 20	163
Female	57%	93
Mean BMI All Patients (kg/m^2^)	28.5 ± 6.7	131
Mean BMI Obese (BMI > 30)	34.5 ± 6.2	83
Mean BMI Non-obese (BMI < 30)	24.6 ± 3.3	80
History of GI Bleed	26%	40
History of Gastroparesis	0.70%	1

The primary outcome was the mean GTT. Analysis of the mean GTT, including and excluding the four patients whose pill did not pass out of the stomach, were not significantly different, and thus, we report values excluding those four patients. The mean GTT (n = 159) was 35 + 49 minutes, median 19, range: 1 - 383. Linear regression did not demonstrate a relationship between GTT and albumin (p = 0.06), HbA1c, or estimated glomerular filtration rate (eGFR) (p = 0.88) as continuous variables, or CKD stage (Kruskal-Wallis p = 0.48).

Table 3 demonstrates that there were no statistically significant differences in GTT or SBTT between the following categorical groups: CKD 0 vs. CKD 2-5, albumin > 3.0 vs. < 3.0, clinical diagnosis of DM status, HbA1c > 6.5% vs. < 6.5%, and obesity.

**Table 3:** PillCam Transit Times Stratified by Patient Demographics (Minutes + Standard Deviation)* *Excludes four patients whose pill never exited the stomach. Numbers for each group listed next to transit times. Clinical and/or transit time data was not available for all patients in the charts reviewed. ** No relationship between the stage of CKD and gastric transit time (Kruskal-Wallis, p = 0.48) *** No relationship between the stage of CKD and small bowel transit time (Kruskal-Wallis, p = 0.13) BMI: body mass index; CHF: congestive heart failure; CKD: chronic kidney disease; n: number; ns: not significant

	Gastric Transit Time	p-value	Small Bowel Transit Time	p-value
Albumin				
> 3 gm/dl	37 ± 53 (n = 123)		237 ± 81.4 (n = 98)	
< 3 gm/dl	27 ± 30 (n = 36)	p = 0.3	240.6 ± 111.4 (n = 26)	p = ns
> 2 gm/dL	37.7 ± 52.2 (n = 109)		227.9 ± 78.9 (n = 81)	
< 2 gm/dL	16.7 ± 20.3 (n = 7)	p = 0.8	245 ± 214.4 (n = 4)	p = ns
No CKD	47.8 ± 78.4 (n = 102)		246.6 ± 83.7 (n = 74)	
CKD Stages 2-5	44.3 ± 82.7 (n = 48)	p = 0.3	225 ± 97.3 (n = 38)	p = ns
CKD 2	11 ± 1.4 (n = 2)		182 ± 43	
CKD 3	27.8 ± 22 (n = 23)		244 ± 107	
CKD 4	23.7 ± 28 (n = 10)		254 ± 87	
CKD 5	34.7 ± 39 (n = 11)**		164 ± 75***	
BMI > 30	33.2 ± 41.4 (n = 81)		244.89 ± 88.0 (n = 69)	
BMI < 30	36.6 ± 56.2 (n = 78)	p = 0.8	229.09 ± 88.0 (n = 55)	p = ns
HbA1c > 6.5%	37 ± 27 (n = 13)		254 ± 105 (n = 11)	
HbA1c < 6.5%	40 ± 53 (n = 38)	p = 0.1	224 ± 94 (n = 27)	p = ns
Systolic CHF	45 ± 28 (n = 19)		246 ± 86 (n = 16)	
No systolic CHF	35 ± 53 (n = 125)	p = 0.003	238 ± 90 (n = 96)	p = ns

We conducted limited posthoc analyses and did not find significant differences in mean GTT when the threshold for low albumin was < 2.5 mg/dL (n = 26) or < 2.0 mg/dL (n = 7) or when we compared the mean GTT for patients with CKD (Stage 4 or 5) (29 minutes) to those without CKD (26 minutes). Data for SBTT was available for 124 patients. The mean SBTT was 238 minutes + 88. Similarly, there was no relation between SBTT and albumin, any CKD, CKD 0 vs CKD 5, DM status, or BMI. Importantly, while the mean gastric transit time was 35 minutes, the median was 19, meaning that the GTT was < 20 minutes for 50% of the population and it was < 76 minutes for 90% (Table 3). 

There were 14 patients with an HbA1c ≥ 6.5% and 38 with HbA1c ≤ 6.4%. There was no significant difference (p = 0.9) between the mean GTT for those with a normal HbA1c (43 minutes) and those with an elevated HbA1c (37 minutes) nor were there significant differences in GTT or SBTT in relation to a history noted in the chart documenting the presence of systolic or diastolic congestive heart failure (Table 3) or smoking status.

## Discussion

This study found that the median GTT was 19 minutes, and to our knowledge, this is the first to report that GTT, per PillCam data, is not prolonged in those with CKD or end-stage renal disease. Uremia in CKD has been associated with gastritis, disruption of the microbiome, and dysmotility disorders [15]. A single magnetic resonance imaging (MRI) study found that gastric emptying was 20 minutes longer in 12 patients with CKD Stage 4/5, but that data conflicts with our findings that GTT via PillCam was only six minutes longer among 21 patients in the CKD 4/5 group [15]. We are not aware of other studies reporting GTT results by PillCam data among patients with CKD, and our CKD 4/5 cohort is larger than the MRI study. The conflicting results between the MRI study (n = 12) and our PillCam study (n = 21), as well as the counterintuitive finding that GTT was shorter among our patients with CKD 4/5, lead us to the following conclusion: to date, there is no conclusive data to support the theory that GTT is longer among patients with any CKD. Our sample size was too small to conduct a meaningful analysis of CKD subgroups so we focused our presentation on the categories of CKD Stage 0 vs CKD Stages 2 - 5. In addition, our results are consistent with prior studies that GTT was not related to other factors that could be associated with neuropathy or longer transit times, such as low albumin, diabetes, and obesity [16-18]. Obesity was not associated with prolonged scintigraphic gastric emptying after a semisolid meal, and our data shows that the same is true for VCE [19].

The PillCam is a non-invasive wireless camera device developed to visualize the entire small intestine mucosa, and the principal indication for this study was an obscure GI bleed [20]. Several devices have been developed and approved by the Food and Drug Administration (FDA); however, the PillCam SB3 is most widely used in the United States (US) [21]. GTT and SBTT are obtained via analysis of the video images. GTT is defined as the time from ingestion of the capsule to the time of the first visualization of the duodenum, and the SBTT is the time from the pyloric passage until the first ileocecal image. The mean GTT in our study measured through the VCE was similar to previously reported values [22-24]. Additionally, while one study found that diabetics classified by chart review (n = 40) had similar GTT and SBTT compared to non-diabetics (n = 87), and the transit times were similar to those reported here, they did not report findings concerning HbA1c values as in this study [23]. An analysis of 89 subjects found no association between transient times and age or BMI [24]. 

It is important to understand that GTT and SBTT, as documented by the wireless motility capsule (WMC), will differ significantly compared to times recorded via the video capsule. Rather than video confirmation, WMC utilizes a pH sensor; the transition to the intestine is documented by a rise in pH and is performed after a solid meal which results in a longer GTT. During the fasting state, phase III migratory motor complexes sweep the gastrointestinal tract, while after a meal the migratory motor complex is delayed until gastric emptying of solids is completed [25]. Specifically, the mean GTT was 208 minutes via a WMC and 43 minutes via a VCE, and the respective SBTT was 5.15 and 4.15 [26]. In a comparative study, healthy subjects underwent the gold standard of whole gut scintigraphy and simultaneous WMC. The GTT obtained with the WMC was 190 ± 54.0 minutes which correlated with the Tc-99 radiolabel retained in the stomach at 120 and 240 min [10, 27]. This GTT mean value differs from the VCE; in non-fasting conditions, the GGT value by the VCE might underestimate the real GTT, but our patients were all in a fasting state.

Historically, GTT as not been related to the type of bowel prep (n = 186). For liquid diet, sodium phosphate, and polyethylene glycol, the respective mean GTT was 25, 34.7, and 35 minutes; the respective SBTT times were 264.4, 296.7, and 291.3 minutes [28].

The strengths of this study are that it was, to our knowledge, the first to evaluate transit times in relation to renal disease and hypoalbuminemia. The similarity between our transit times and prior reports suggests that ours are robust and generalizable to a generally healthy patient population. The size of our cohort is also similar to prior reports. Limitations include only 10 patients being on any kind of narcotic, 13/38 diabetic patients had an HbA1c > 6.5%, and an inability to collect demographic data on outpatients beyond their age and the indication for the test, thus decreasing the sample size for the subgroup analyses. As with any retrospective review, it is also possible that useful clinical information was omitted from the chart. We excluded four patients whose PillCam was lodged in the stomach, but as stated above, the analysis including and excluding those four patients was not different. Additionally, none of the patients had a comorbid condition identified by chart review that could explain this occurrence.

## Conclusions

In summary, this analysis found that the mean GTT was 35 minutes, the median time was 19 minutes for 50% of the cohort, and < 76 minutes for 90% of the cohort. These data support historical findings that endoscopic procedures can be safely conducted in the majority of patients within 60 minutes of ingesting liquids and that clinicians should not inconvenience patients who have not eaten for hours and/or endured a colonoscopy prep or busy endoscopists by delaying endoscopic procedures. To our knowledge, this is the first study to demonstrate that the presence of any stage of kidney disease was not associated with delayed GTT, as documented by the PillCam. Additionally, as the PillCam is performed in a fasting state, this data is generalizable to patients preparing for endoscopic procedures.
